# Systematics of the Oswaldoi Complex (*Anopheles, Nyssorhynchus*) in South America

**DOI:** 10.1186/1756-3305-6-324

**Published:** 2013-11-12

**Authors:** Freddy Ruiz-Lopez, Richard C Wilkerson, David J Ponsonby, Manuela Herrera, Maria Anice Mureb Sallum, Ivan Dario Velez, Martha L Quiñones, Carmen Flores-Mendoza, Dave D Chadee, Joubert Alarcon, Joubert Alarcon-Ormasa, Yvonne-Marie Linton

**Affiliations:** 1 Department of Entomology, National Museum of Natural History, Smithsonian Institution, Museum Support Center, Suitland, MD 20746, USA; 2 Programa de Estudio y Control de Enfermedades Tropicales (PECET), Facultad de Medicina, Universidad de Antioquia, Medellín, Colombia; 3 Walter Reed Biosystematics Unit, Smithsonian Institution, Museum Support Center, Suitland, MD 20746, USA; 4 Department of Geographical and Life Sciences, Canterbury Christ Church University, Kent, UK; 5 Facultad de Medicina, Universidad Nacional de Colombia, Bogotá, Colombia; 6 Departamento de Epidemiologia, Faculdade de Saúde Pública, Universidad de São Paulo, São Paulo, SP, Brazil; 7 Naval Medical Research Unit (NAMRU-6), Lima, Peru; 8 Department of Life Science, Faculty of Science and Technology, University of the West Indies, St. Augustine Campus, West Indies, Trinidad and Tobago; 9 Servicio Nacional de Control de Enfermedades Transmitidas por Vectores Artrópodos, Ministerio Salud Publica, Guayaquil, Ecuador; 10 Walter Reed Army Institute of Research, 503 Robert Grant Avenue, Silver Spring, MD 20910, USA

**Keywords:** *Anopheles oswaldoi* species complex, *An. oswaldoi* s.s., *An. oswaldoi* A, *An. oswaldoi* B, *An.* sp. nr. *konderi*, *COI* barcoding, ITS2

## Abstract

**Background:**

Effective malaria control relies on accurate identification of those *Anopheles* mosquitoes responsible for the transmission of *Plasmodium* parasites. *Anopheles oswaldoi* s.l. has been incriminated as a malaria vector in Colombia and some localities in Brazil, but not ubiquitously throughout its Neotropical range. This evidence together with variable morphological characters and genetic differences supports that *An. oswaldoi* s.l. compromises a species complex. The recent fully integrated redescription of *An. oswaldoi* s.s. provides a solid taxonomic foundation from which to molecularly determine other members of the complex.

**Methods:**

DNA sequences of the Second Internal Transcribed Spacer (ITS2 - rDNA) (n = 192) and the barcoding region of the *Cytochrome Oxidase I* gene (*COI* - mtDNA) (n = 110) were generated from 255 specimens of *An. oswaldoi* s.l. from 33 localities: Brazil (8 localities, including the lectotype series of *An. oswaldoi*), Ecuador (4), Colombia (17), Trinidad and Tobago (1), and Peru (3). *COI* sequences were analyzed employing the Kimura-two-parameter model (K2P), Bayesian analysis (MrBayes), Mixed Yule-Coalescent model (MYC, for delimitation of clusters) and TCS genealogies.

**Results:**

Separate and combined analysis of the *COI* and ITS2 data sets unequivocally supported four separate species: two previously determined (*An. oswaldoi* s.s. and *An. oswaldoi* B) and two newly designated species in the Oswaldoi Complex (*An. oswaldoi* A and *An.* sp. nr. *konderi*). The *COI* intra- and inter-specific genetic distances for the four taxa were non-overlapping, averaging 0.012 (0.007 to 0.020) and 0.052 (0.038 to 0.064), respectively. The concurring four clusters delineated by MrBayes and MYC, and four independent TCS networks, strongly confirmed their separate species status. In addition, *An. konderi* of Sallum should be regarded as unique with respect to the above. Despite initially being included as an outgroup taxon, this species falls well within the examined taxa, suggesting a combined analysis of these taxa would be most appropriate.

**Conclusions:**

Through novel data and retrospective comparison of available *COI* and ITS2 DNA sequences, evidence is shown to support the separate species status of *An. oswaldoi* s.s., *An. oswaldoi* A and *An. oswaldoi* B, and at least two species in the closely related *An. konderi* complex (*An.* sp. nr. *konderi*, *An. konderi* of Sallum). Although *An. oswaldoi* s.s. has never been implicated in malaria transmission, *An. oswaldoi* B is a confirmed vector and the new species *An. oswaldoi* A and *An.* sp. nr. *konderi* are circumstantially implicated, most likely acting as secondary vectors.

## Background

Species complexes are relatively common in the family Culicidae [1], and several Neotropical *Anopheles,* including some vector species, are known to comprise isomorphic species. Within the Oswaldoi Group alone (*Anopheles*, subgenus *Nyssorhynchus*), seven of the 15 formally recognised species comprise complexes [2-11]. *Anopheles oswaldoi* (Peryassú) is one such taxon. It is thought to comprise a species complex in Brazil [4,9,12-16] and Colombia [8,13,17] and has been implicated in malaria transmission in some parts of its range [11-13], yet its taxonomic and vectorial status elsewhere in South America remains unclear. A comprehensive revision of the taxonomy and current distribution of *An. oswaldoi* is given in Motoki *et al.*[12].

Several studies have provided evidence for genetic variation in *An. oswaldoi*. Firstly, Marrelli *et al*. [14] analyzed ITS2 sequences from seven populations of *An. oswaldoi* s.l. and determined four geographic groups, as follow: Group I from Acre, Amazonas and Rondônia (Brazil), Group II from Ocamo (Venezuela) and Amapá (Brazil), Group III from Espírito Santo (type locality), Brazil and Group IV from Yurimaguas, Peru. Subsequently, Ruiz *et al*. [17] revised these groupings and determined that Group II corresponded to *An. oswaldoi* B from Putumayo, Colombia [13], and that Group IV from Yurimaguas, Peru, was actually *An. benarrochi* B [17], a newly recognized species of the subgenus *Nyssorhynchus*[17,18]. The identification of Group III (GenBank: AF055072) from the type locality of Espírito Santo in Brazil was later corrected to that of *An. evansae* (Bréthes) [4]. The misidentification resulted from the incorrect use of polymorphic characters in the wing (humeral pale spot) and second hindtarsal segment (basal dark band); characters that overlap between *An. oswaldoi* and *An. evansae* in currently available taxonomic keys [11]. The true identity of Group I remains unclear.

Scarpassa and Conn [16] sequenced a long fragment of *COI* from 45 *An. oswaldoi* s.l. from four populations from Brazil (Acre, Amazonas, Rondônia and Pará). Parsimony analysis revealed four distinct groups: Group I, Acre (Sena Madeira) and Rondônia (São Miguel); Group II, Rondônia (São Miguel); Group III, Pará (Moju), and Group IV from Acre (Sena Madureira) and Coari (Amazonas). Although the authors tentatively suggested that Group I may be *An. oswaldoi* s.s*.* and Group IV may be *An. konderi* Galvão and Damasceno, based primarily on geographic origin, they lacked certainty in assigning taxonomic names to these phylogenetic lineages.

The taxonomic confusion between *An. oswaldoi* and *An. konderi* is not new. *Anopheles konderi* was originally described from specimens collected in the Solimões River at Coari, Amazonas, Brazil in 1942 [19]. Soon after, Lane [20] reduced it to a junior synonym of *An. oswaldoi*, where it remained until its re-elevation to separate species status in 2004 [21]. Isoenzyme analysis of 20 loci in three populations of purported *An. oswaldoi* and *An. konderi* from the Brazilian Amazon (Coari, Amazonas (*An. konderi*); São Miguel, Rondônia (*An. oswaldoi* and *An. konderi*) and Sena Madureira, Acre (*An. oswaldoi* and *An. konderi*)) revealed no significant differences between populations, and led the author to question whether *An. oswaldoi* and *An. konderi* were indeed truly separate species, or whether ongoing introgression between the two species would explain this low level of variation [22]. Recently, Motoki *et al.*[23] using *COI*, *white* and ITS2 DNA sequences confirmed that morphologically identified specimens of *An. konderi* comprised at least three species in the Amazonian region. Unfortunately, none of these recent studies (including the redescription [21]) have examined specimens from the type locality, thus the true identity of *An. konderi* s.s. remains unclear.

The same was true for *An. oswaldoi* s.s. until a recent redescription of the species was undertaken based on progeny broods collected from the type locality of Espírito Santo, Brazil [12]. This study included morphological data for immature stages (fourth-instar larvae and pupae) and adults of both sexes, as well as corresponding ITS2 DNA sequence. As a holotype was not designated for *An. oswaldoi* s.s. when it was first described by Peryassú in 1922, a lectotype was chosen from the type series [12]. This study provided a solid taxonomic platform from which to attempt to further determine the component members of the Oswaldoi Complex. Comparison of DNA sequences of *An. oswaldoi* s.s*.*[12] confirms that neither Marrelli *et al*. [14] (ITS2), nor Scarpassa and Conn [16] (*COI*) included “true” *An. oswaldoi* in their studies. To date, *An. oswaldoi* s.s*.* has only been confirmed in the Brazilian States of Espírito Santo, Rio de Janeiro and São Paulo in Brazil [12,24]. Pinault and Hunter [25] recently reported three *COI* sequences of *An. oswaldoi* from Ecuador (GenBank: JN412831-33), however these sequences were misidentified, sharing high similarity with those of *An. rangeli* (GenBank: HM022390-94). Based on morphology and ITS2 sequences, Sallum *et al*. [9] detected at least two cryptic species in *An. oswaldoi* s.l. collected in the state of Acre (Brazil), both of which differ from *An. oswaldoi* s.s.

Given the evidence above, there is no doubt that *An. oswaldoi* comprises a species complex in Latin America. The objectives of this study were to ascertain the taxonomic status and relative distribution of the component members of the *An. oswaldoi* species complex in Brazil, Colombia, Ecuador, Peru, Trinidad and Tobago and Venezuela using ITS2 and *COI* barcodes, and to correlate this information with previously documented vector incrimination studies of *An. oswaldoi* s.l. across its range.

## Methods

### Specimens

A total of 255 specimens of *Anopheles oswaldoi s.l*. from 33 localities in five countries in the Neotropics (Brazil, Colombia, Ecuador, Peru and Trinidad and Tobago) were used in this study (see Table 1 for georeferenced locality data). Co-ordinates were converted to decimal degrees [26] and the distribution data is available in Mosquito Map (http://www.mosquitomap.org). Samples of *Anopheles konderi* s.l. were collected near Macapá, Amapá, Brazil and identified on the basis of the male aedeagus by MAMS. To avoid confusion, these are referred to herein as *An. konderi* of Sallum. All specimens used in this study were collected or provided by the authors of this study.

**Table 1:** **Origin and georeferences of *An. oswaldoi *s.l. specimens used in this study, showing relative numbers of *COI *(n = 110) and ITS2 (n = 192) sequences obtained from 255 specimens have been used**

**Country**	**State and exact locality**	**n =**	***COI***	**ITS2 (H)**	**Latitude**	**Longitude**
Brazil	Amazonas, Tefé	1	1	1 (III)	-03.3207	-64.7236
	Espirito Santo, Jaguaré, Fazenda Marianelli	10	3	10 (I)	-19.0348	-39.9485
	Mato Grosso, Peixoto de Azevedo	42	42	14 (II, IV-IX)	-10.2257	-54.9862
	Rio de Janeiro, Morro de Panela	2	2	-	-22.9678	-43.3-415
	Rondônia, Ariquemes	3	3	-	-09.9136	-63.0440
	Rondônia, Costa Marques	3	3	-	-12.4156	-64.2215
	São Paulo, Pariquera-Açu	2	2	2 (I)	-24.9875	-47.9561
	São Paulo, Pariquera-Açu	1	1	-	-24.7096	-47.8839
Colombia	Amazonas, Kilometro 12	1	1	1 (III)	-04.1159	-69.9522
	Antioquia, Nechí, Mala Noche	28	12	28 (X)	08.1101	-74.7671
	Caquetá, Peñas Coloradas	2	2	1 (XII)	00.8699	-73.8419
	Meta, Granada, Morichal	2	2	-	03.5372	-73.7009
	Norte de Santander, Tibú	1	1	1 (X)	08.6403	-72.7371
	Putumayo, Agua Negra	14	-	14 (XII)	00.7494	-75.3834
	Putumayo, Cecilia Cocha	5	-	5 (XII)	00.1158	-74.9781
	Putumayo, La Apaya	6	-	6 (XII)	00.7494	-75.3833
	Putumayo, Pto. Asís, Cecilia Cocha	24	-	24 (XII)	00.1158	-74.9781
	Putumayo, Pto. Asís, La Manuela	4	4	-	00.5133	-76.4992
	Putumayo, Pto. Leguizamo, Bella Vista	8	-	8 (XII)	00.7494	-75.3833
	Putumayo, Pto. Leguizamo, El Salado	9	-	9 (XII)	00.2108	-74.8036
	Putumayo, Pto. Leguizamo, La Quebradita	4	4	2 (XII)	00.5133	-76.4992
	Putumayo, Pto. Leguizamo, Lagarto Cocha	39	-	39 (XII)	00.2108	-74.8036
	Putumayo, Pto. Leguizamo, Puntales	13	-	13 (XII)	00.4272	-74.3986
	Putumayo, Pto. Leguizamo, Tukare	1	1	1 (XII)	00.5133	-76.4992
	Putumayo, Pto. Nariño	2	-	2 (XII)	00.7494	-75.3833
Ecuador	Orellana, Coca, Cañon de los Monos	2	2	-	-00.3434	-77.0070
	Orellana, Coca, Guamayacu	4	4	-	00.1300	-77.2313
	Orellana, Coca, Juan Montalvo	7	7	-	-00.4725	-76.9914
	Orellana, Tiputini	2	2	-	-00.6381	-76.1450
Peru	Loreto, Iquitos	1	1	-	-03.7561	-73.2706
	Loreto, Rio Putumayo	1	1	-	-04.2325	-74.2179
	Madre de Dios, Davila	1	1	1 (XIII)	-11.7669	-70.8119
Trinidad and Tobago	Valencia, St. Andrew/St. David	10	8	10 (XI)	10.6447	-61.0936

Sequences generated per country include: Brazil (27 ITS2, 57 *COI*), Colombia (154 ITS2, 27 *COI*), Ecuador (15 *COI*), Peru (1 ITS2, 3 *COI*) and Trinidad and Tobago (10 ITS2, 8 *COI*). H: ITS2 haplotype number.

### Molecular analysis

DNA was extracted using the DNeasy® Blood and Tissue Kit (QIAgen®, USA) on the automated BioSprint 96® robotic platform. The ITS2 region was amplified for 192 samples from 21 localities using the published primers of Collins and Paskewitz [27] and following the protocol in Linton *et al*. [28]. DNA barcodes [29] were amplified from 110 individuals from 24 localities using the universal barcoding primers developed by Folmer *et al*. [30] and the protocol of the Mosquito Barcoding Initiative, expressly listed in Ruiz *et al*. [31].

Sequencing reactions were carried out in both directions using the Big Dye Terminator Kit® on an ABI3770 automated sequencer (PE Applied BioSystems®, Warrington, England). Sequence chromatograms were edited using Sequencher™ v. 4.8 (Genes Codes Corporation®, Ann Arbor, MI). Sequences were aligned automatically in Mafft Pro 5.5.7 (http://www.geneious.com) or using MacClade v. 4.06 [32]. Basic Local Alignment Search Tool (BLAST) searches (http://blast.ncbi.nlm.nih.gov) were carried out to correlate our sequences with those publicly available in GenBank.

To assess population-level genealogies, *COI* sequences were analyzed using TCS v. 1.21 [33]. A connection limit of 95 % was adopted to investigate whether *An. oswaldoi* formed a single “meta-population” (reflected by a single network) or is comprised of separate species in South America (reflected by the formation of two or more independent networks).

A *COI* data matrix was generated in MEGA v. 5 [34]. *COI* sequences were grouped according to the results of the TCS analysis, and intra- and inter-group genetic distances compared using Kimura’s 2-Parameter distance (K2P) algorithm [35]. Phylogenetic analysis was carried out on the separate and combined ITS2 and *COI* data sets. MrModeltest v. 2.3 [36] was used to choose the best evolutionary model for these regions separately using the Akaike Information Criterion (AIC) search. A partitioned Bayesian analysis (by DNA region) was subsequently performed using MrBayes v. 3.1.2 [37], available online (http://cbsuapps.tc.cornell.edu/mrbayes.aspx).

The analysis in MrBayes ran for 10 million generations with two parallel searches using three heated and one cold Markov chain. The first 5 million generations were discarded as burn-in. Support for this Bayesian tree was conducted by generating a maximum parsimony (MP) tree in PAUP v. 4.0b10 [38] for both ITS2 and *COI*, and bootstrapping [39], using a heuristic search, simple stepwise addition, TBR branch swapping and 1000 bootstrap replicates.

Unique *COI* haplotypes were further analysed using MYC [40,41] for delimitation of *COI* clusters. This method optimizes a threshold age that corresponds to the shift from coalescent to species diversification [42] branching processes and calculates the number of resulting independent entities. The likelihood of the null model that all samples belong to a single species is compared to that of the alternative hypothesis where separate coalescent groups are nested within the species tree. Confidence limits correspond to threshold values ±2 logL units around the ML estimate. This analysis was conducted on Bayesian consensus trees and each tree was converted to ultrametric using penalized likelihood as implemented in r8s v.1.7 [43], with the optimal smoothing parameter selected by cross-validation of values between 0.01 and 1000. FigTree v. 1.2.1 [44] was used to edit all trees generated.

## Results

Specimens of *An. oswaldoi s.l.* (n = 255) were obtained from 33 localities in five South American countries as follow (Table 1): Brazil (n = 64), Colombia (n = 163), Ecuador (n = 15), Peru (n = 3) and Trinidad and Tobago (n = 10). From these mosquitoes, 192 ITS2 sequences were generated and a subset of 110 of these, including four specimens from the redescription [12], were also barcoded. The full data sets by country follow: Brazil (27 ITS2; 57 *COI*, GenBank: KF809034-078, KF809121-132), Colombia (154 ITS2; 27 *COI*, GenBank: KF809079, KF809085-091, KF809093-096, KF809100, KF809102, KF809105, KF809109-119, KF809133), Ecuador (15 *COI*, GenBank: KF809081-084, KF809099, KF809103, KF809108, KF809120, KF809135-139, KF809142-143), Peru (1 ITS2; 3 *COI*, GenBank: KF809134, KF809140-141) and Trinidad and Tobago (10 ITS2; 8 *COI,* GenBank: KF809080, KF809092, KF809097-098, KF809101, KF809104, KF809106-107, KF809128). COI sequences of *An. konderi* sensu Sallum (n=4) are available in GenBank under accessions KF809030-033.

### ITS2 sequences of *An. oswaldoi* s.l

ITS2 sequences were generated for 192 specimens of *An. oswaldoi* s.l. (Table 1). The overall alignment (544 bp, after primer trim) revealed 13 unique ITS2 haplotypes (GenBank: KC970065-77), labelled HI-HXIII (Table 2). The ITS2 sequences ranged in length from 530 bp in Haplotype I (H1) (Espírito Santo and São Paulo) to 540 bp in haplotypes HVI-IX from Mato Grosso in Brazil (Table 2). Thirty-two variable bases were noted (5.9 %), with the variation concentrated toward the second half (3′ end) of the ITS2 fragment between aligned nucleotides 207–494. Indels (insertions/deletions) were observed at bases 337–338, 344–345, 365–369, 408–409, 416–419, and 467–468 (Table 2).

**Table 2:** **Comparative alignment and size differentials of the 13 unique ITS2 haplotypes detected in 192 specimens of *An. oswaldoi *s.l. from Brazil (BR, n = 27), Colombia (CO, n = 154), Peru (PE, n = 1) and Trinidad and Tobago (TR, n = 10)**

			**2**	**2**	**3**	**3**	**3**	**3**	**3**	**3**	**3**	**3**	**3**	**3**	**3**	**3**	**4**	**4**	**4**	**4**	**4**	**4**	**4**	**4**	**4**	**4**	**4**	**4**	**4**	**4**	**4**	**4**	**4**	**4**
			**0**	**5**	**1**	**3**	**3**	**3**	**4**	**4**	**4**	**6**	**6**	**6**	**6**	**6**	**0**	**0**	**0**	**1**	**1**	**1**	**1**	**1**	**5**	**6**	**6**	**6**	**7**	**7**	**7**	**7**	**8**	**9**
**Haplptypes**	**n**	**Spicemen origen**	**7**	**9**	**0**	**2**	**7**	**8**	**2**	**4**	**5**	**5**	**6**	**7**	**8**	**9**	**4**	**8**	**9**	**2**	**6**	**7**	**8**	**9**	**5**	**1**	**7**	**8**	**0**	**3**	**4**	**7**	**6**	**4**
HI	12	Espirito Santo/São BR	A	C	A	A	-	-	A	-	-	A	-	-	A	C	C	A	G	C	-	-	-	-	C	A	-	-	G	-	-	G	A	C
HII	1	Mato Grosso, BR	A	C	T	C	-	-	A	-	-	A	C	C	A	T	C	A	G	A	A	G	A	A	C	A	-	-	G	G	C	G	A	A
HIII	2	Amazonas, BR & Amazonas, CO	G	C	T	C	-	-	A	-	-	A	T	C	A	T	C	A	G	A	A	G	A	A	C	A	-	-	G	G	C	G	G	A
HIV	4	Mato Grosso, BR	G	C	T	C	-	-	A	-	-	A	C	C	A	T	C	A	G	A	A	G	A	A	C	A	-	-	G	G	C	G	G	A
HV	3	Mato Grosso, BR	G	C	T	C	-	-	A	-	-	A	C	C	A	T	C	A	G	A	A	G	A	A	C	A	-	-	G	G	C	G	A	A
HVI	1	Mato Grosso, BR	G	C	T	A	-	-	A	A	G	A	C	C	A	T	C	A	G	A	A	G	A	A	C	G	-	-	G	G	C	G	A	C
HVII	3	Mato Grosso, BR	G	C	T	A	-	-	A	A	G	A	T	C	A	T	C	A	G	A	A	G	A	A	C	A	-	-	G	G	C	G	G	A
HVIII	1	Mato Grosso, BR	G	C	T	A	-	-	A	A	G	A	C	C	A	T	C	A	G	A	A	G	A	A	C	A	-	-	G	G	C	G	A	C
HIX	1	Mato Grosso, BR	G	C	T	A	-	-	A	A	G	A	T	C	A	T	C	A	G	A	A	G	A	A	C	A	-	-	C	G	C	G	A	C
HX	29	Antoquia & Norte de Santander, CO	A	T	T	C	C	A	A	-	-	-	-	-	-	-	A	G	G	C	-	-	-	-	C	A	T	C	C	G	C	A	A	C
HXI	10	St Andrews, TR	A	T	T	C	C	A	A	-	-	-	-	-	-	-	A	G	G	C	-	-	-	-	C	G	T	C	G	G	C	A	A	C
XHII	124	Putumayo & Caqueta, CO	A	T	T	A	C	A	A	-	-	-	-	-	-	-	A	G	G	C	-	-	-	-	C	G	-	-	G	G	C	G	A	C
HXIII	1	Madre de Dios, PE	A	T	A	A	-	-	G	-	-	G	C	C	A	T	C	-	-	A	A	G	A	A	T	A	-	-		G	C	G	G	A

Following primer trim, the ITS2 fragments varied in length from 530–540 bp. In the final 544-bp alignment, 32 sites were found to be variable. H1 includes specimens of the lectotype series of *An. oswaldoi* s.s [12].

Whereas in general all geographical areas were represented by unique ITS2 haplotypes, the 14 specimens of *An. oswaldoi* from Mato Grosso (Brazil) showed seven unique haplotypes (HII, HIV - HIX), varying in length between 538 bp and 540 bp (Table 2). Specimens from Putumayo, Colombia (n = 123) and Caquetá, Colombia (n = 1) shared the same ITS2 haplotype (HXII) and one specimen from Amazonas, Colombia shared the same haplotype with the single specimen sequenced from Amazonas, Brazil (HIII) (Table 2). Haplotype H1 comprises specimens from Espírito Santo and São Paulo, including those in the redescription and lectotype series of *An. oswaldoi* s.s*.*[12].

### MtDNA *COI* sequences of *An. oswaldoi* s.l

The mtDNA *COI* barcode sequences (n = 110, 658 bp without primers) revealed 84 unique haplotypes in the 33 localities sampled (Table 1). The amino acid (AA) reading frame starts at the second base of the primer-edited sequences. Protein translations, carried out using the invertebrate mitochondrial code showed no stop codons, indicating that all sequences represent functional protein coding genes.

**Figure 1:**
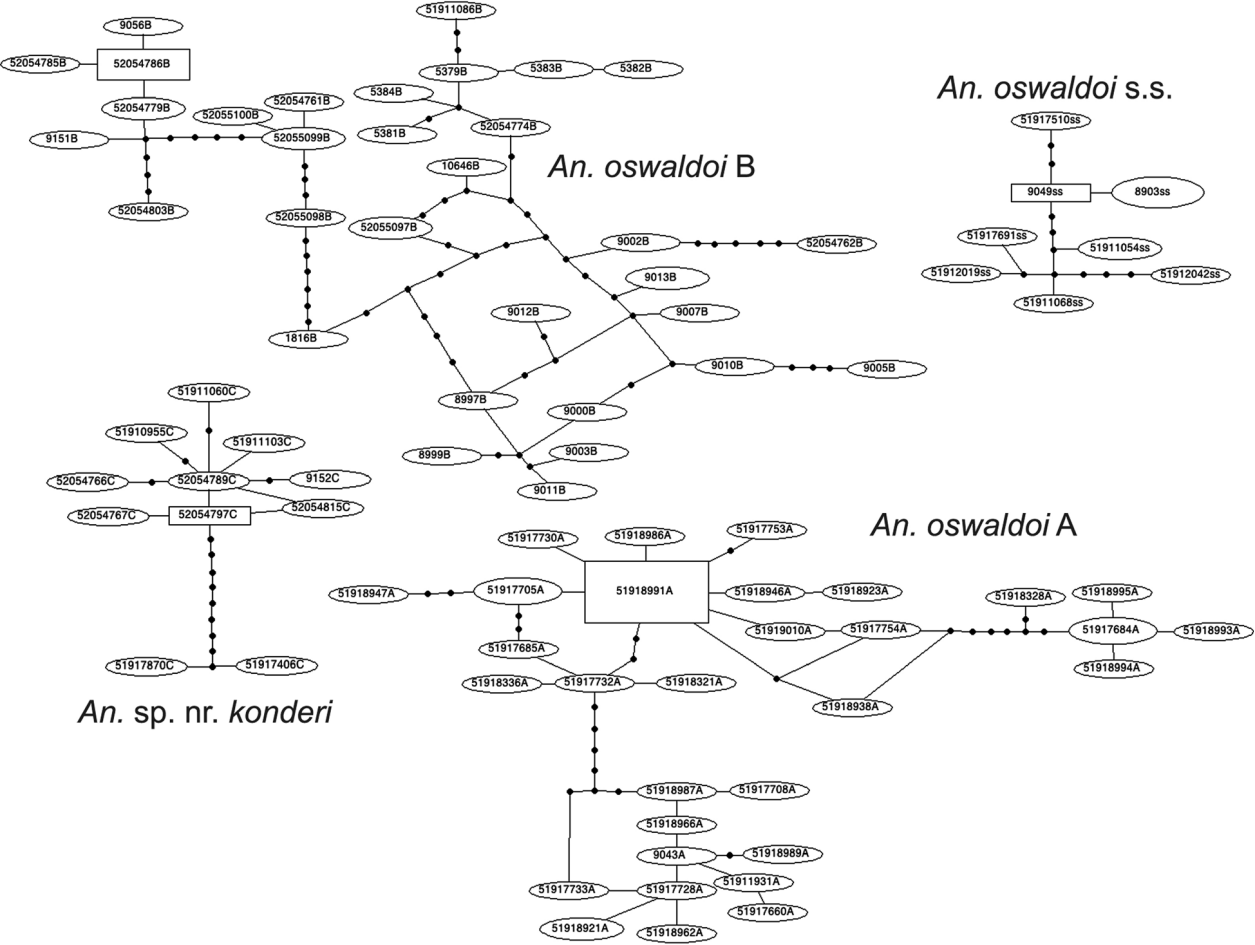
**TCS analysis of *An. oswaldoi *s.l. using *COI *barcode sequences.** Four networks were generated separately; *An. oswaldoi s.s.* from Brazil; *An. oswaldoi* A from Brazil and Colombia; *An. oswaldoi* B from Colombia, Ecuador and Trinidad and Tobago; and *An.* sp. nr*. konderi* from Colombia, Ecuador and Peru.

Meta-population analysis of the *COI* data set using TCS [33] clearly split the data set into four independent networks (Figure 1): *An. oswaldoi* s.s., *An. oswaldoi* A, *An. oswaldoi* B and *An.* sp. nr*. konderi*. This analysis is concordant with the ITS2 haplotypes as follow: *An. oswaldoi* s.s. [12] (haplotype HI), *An. oswaldoi* A (named herein) (HII-IX), *An. oswaldoi* B [18] (HX-XII) and *An.* sp. nr*. konderi* (named herein) (HXIII). Although high levels of genetic divergence (0.020) were noted in *COI* sequences between samples from northern Colombia and Trinidad and Tobago when compared to those from southern Colombia, the TCS analysis showed that these comprised a single species, *An. oswaldoi* B (Figure 1).

Pairwise *COI* sequence comparisons between the groups listed above (TCS network) revealed higher levels of *COI* divergence between them. Intra-specific divergence ranged from 0.007 in *An. oswaldoi* s.s. (n = 12), to 0.02 in *An. oswaldoi* B (n = 41). Inter-specific *COI* sequence divergences ranged from 0.038 in the most closely related species, *An. oswaldoi* s.s. and *An. oswaldoi* B, to 0.064 between *An. oswaldoi* A and *An. oswaldoi* B (Table 3).

**Table 3:** Mean inter- and intra-specific pairwise distances (K2P model)

**Species**	**n =**	**osw s.s.**	**osw A**	**osw B**	**sp. nr. kon**
*An. oswaldoi* s.s.	12	**0.007**			
*An. oswaldoi* A	46	0.057	**0.012**		
*An. oswaldoi* B	41	0.038	0.064	**0.020**	
*An.* sp. nr. *konderi*	11	0.045	0.053	0.056	**0.010**

Analyses of *COI* sequences separated 110 specimens of *Anopheles oswaldoi* s.l. into four groups using the TCS model as calculated by Kimura’s 2-Parameter distance model (K2P) [35].

**Figure 2:**
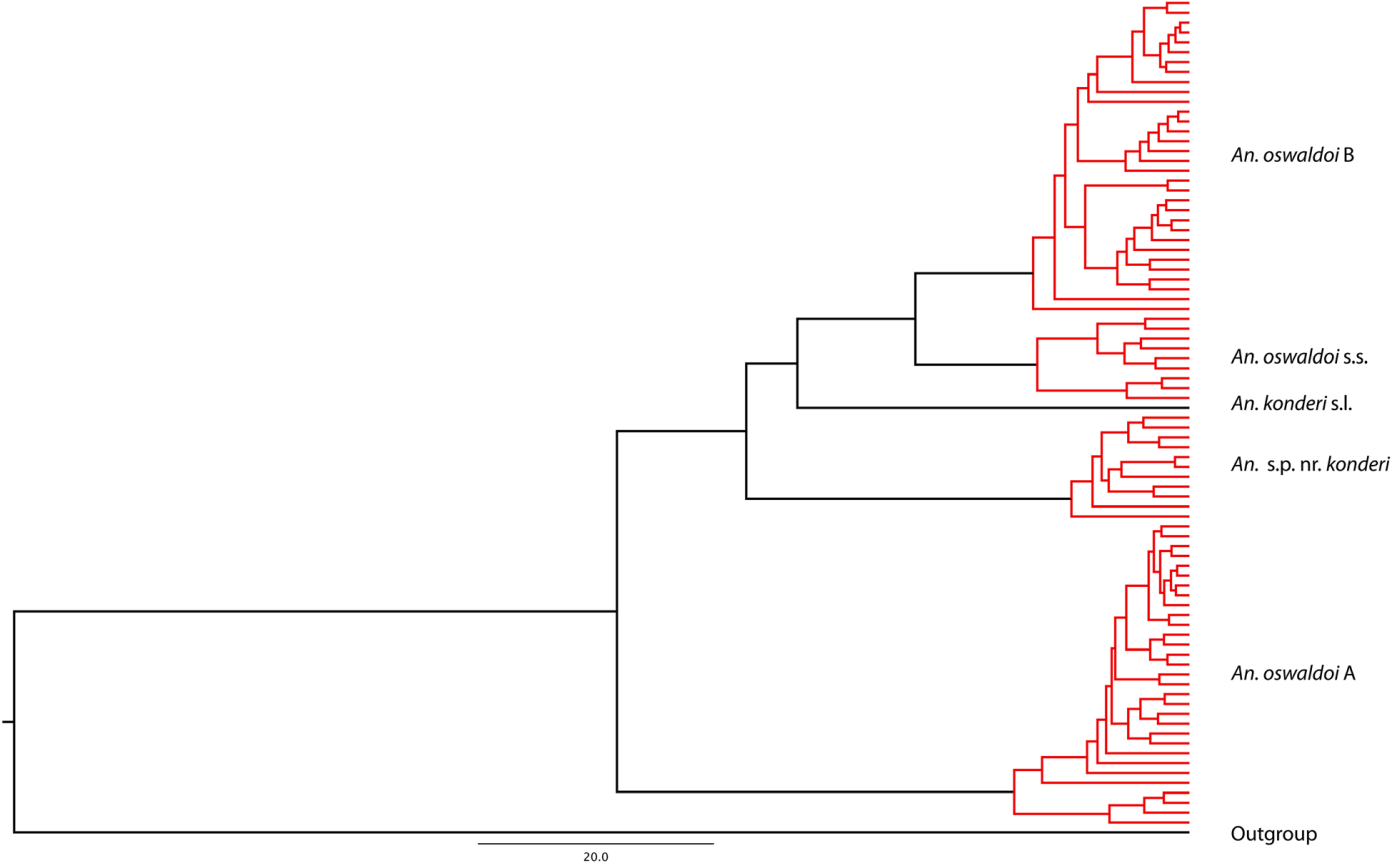
**Mixed Yule-Coalescent model tree of *An. oswaldoi *s.l. from the Neotropical region, using *COI *sequences.** Four groups are clearly delineated with MYC: The *An. oswaldoi* s.s. from Brazil (Espírito Santo, Rio de Janeiro, Rondônia and São Paulo, Brazil); *An. oswaldoi* A from Brazil (Mato Grosso, Rondônia and Tefé), Colombia (Amazonas); *An. oswaldoi* B from Colombia (Antioquia, Caquetá, Meta, Norte de Santander and Putumayo), Ecuador (Orellana) and Trinidad and Tobago (Valencia); *An.* sp. nr*. konderi* from Ecuador (Orellana), Colombia (Caquetá) and Peru (Loreto and Madre de Dios). Outgroup: *An. albitarsis* F and *An. nuneztovari* C.

Four clusters, corresponding to *An. oswaldoi* s.s*.* (Brazil), *An. oswaldoi* A (Brazil and Colombia), *An. oswaldoi* B (Colombia, Ecuador and Trinidad and Tobago) and *An.* sp. nr. *konderi* (Ecuador, Colombia and Peru), were delineated using the MYC model [41] (Figure 2). The MYC model uses only the unique *COI* haplotypes, which detects the transition between species to within-population branching patterns. This analysis also lends support for the separate species status of *An. konderi* of Sallum from Macapá, Amapá, Brazil (GenBank: KF305833), and its close relationship with *An. oswaldoi* s.l.

**Figure 3:**
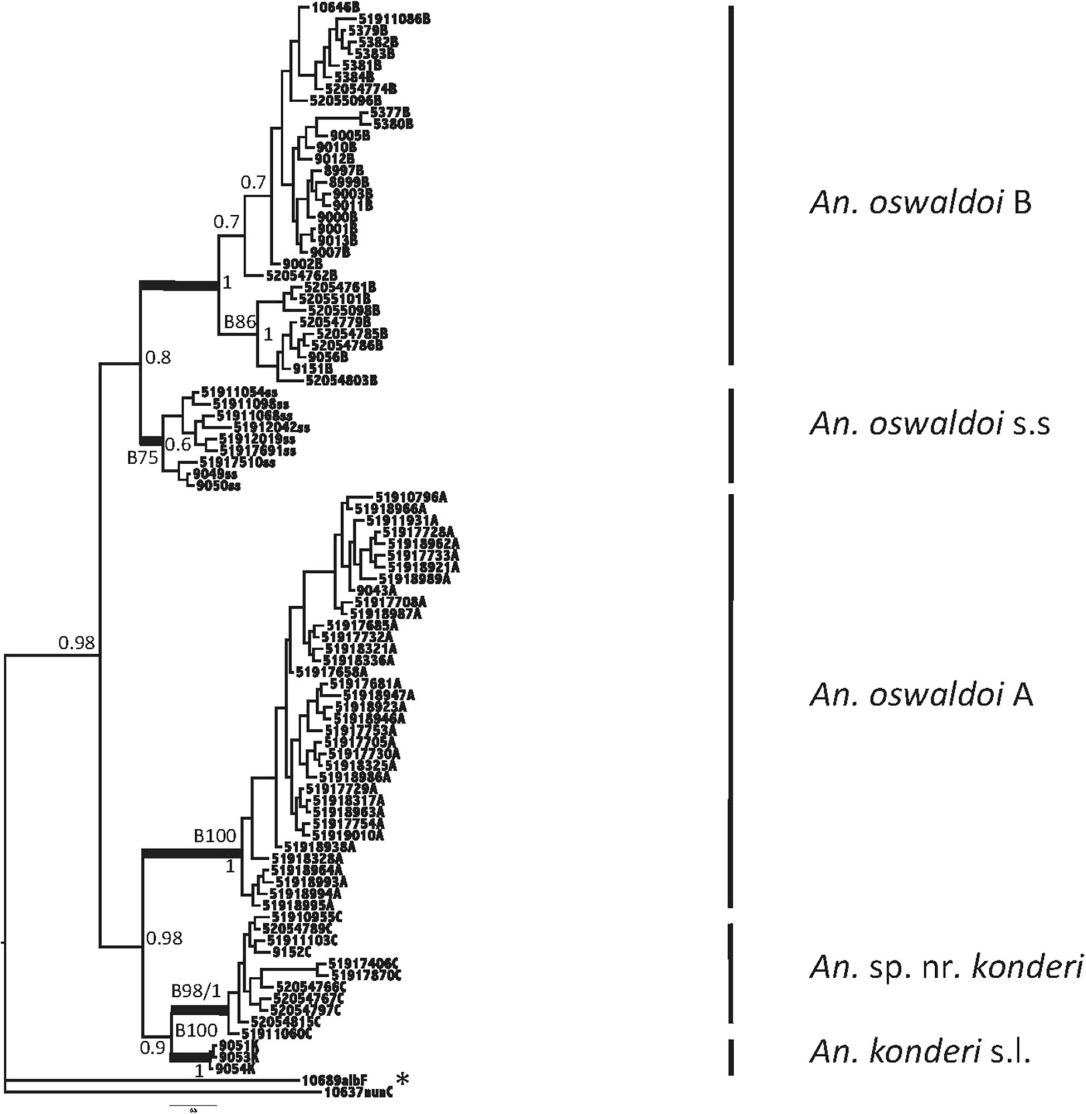
**Combined MrBayes tree of *An. oswaldoi *s.l. using ITS2 and *COI *sequences.** Four groups are clearly defined *An. oswaldoi* s.s. (Espírito Santo, Rio de Janeiro, Rondônia and São Paulo, Brazil); *An. oswaldoi* A (Mato Grosso, Rondônia and Tefé, Brazil and Amazonas, Colombia); *An. oswaldoi* B from Colombia (Antioquia, Caquetá, Meta, Norte de Santander and Putumayo), Ecuador (Orellana) and Trinidad and Tobago (Valencia); and *An.* sp. nr*. konderi* from Ecuador (Orellana), Colombia (Caquetá) and Peru (Loreto and Madre de Dios). B: Bootstrap; P: Posterior probability. *Anopheles konderi* of Sallum from near Macapá, Amapá, Brazil, is clearly differentiated from the other species of the *An. oswaldoi* complex, and appears to sit within the Oswaldoi Group. *Outgroups: *An. albitarsis* F and *An. nuneztovari* C.

The combined analysis of both *COI* and ITS2 sequences using MrBayes [36], with the HKY+I+G (Hasegawa-Kishino-Yano + Invariant Sites + Gamma) model, again strongly confirmed that *An. oswaldoi* s.l. is a complex of at least four species (Figure 3). The posterior probability had a value of one, for three clades (*An. oswaldoi* A, *An. oswaldoi* B and *An.* sp. nr*. konderi*) and 0.6 in *An. oswaldoi* s.s. however, bootstrap values fully confirm these four species (*An. oswaldoi* s.s., *An. oswaldoi* A, *An. oswaldoi* B and *An.* sp. nr*. konderi*) (Figure 3). The *COI* sequence of *An. konderi* of Sallum was found to cluster closely with *An.* sp. nr*. konderi*, but these two species are consistently different in four of their ITS2 bases as follows: one transition in position 361 (G/A), two indels (364 (A/-) and 365 (T/-)), and one transversion (449 T/C) (data not shown).

**Figure 4:**
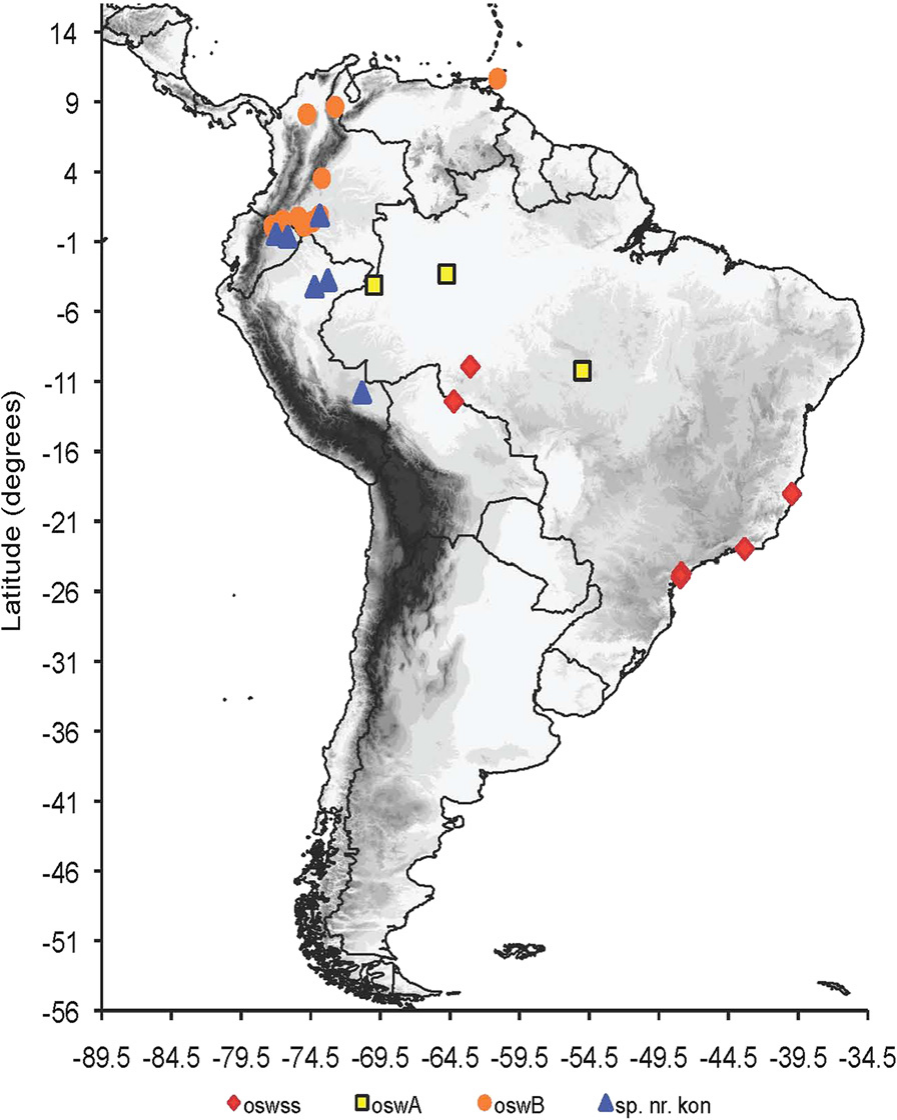
**Map showing the overall distribution of *An. oswaldoi *s.l. in the Neotropical region based on *COI *and ITS2 sequences.** oswss: *An. oswaldoi s.s*. (red) in Brazil; oswA: *An. oswaldoi* A (yellow) in Brazil and Colombia; oswB: *An. oswaldoi* B (orange) in Colombia, Ecuador and Trinidad and Tobago; and sp. nr. kon: *An.* sp. nr*. konderi* (blue) in Colombia, Ecuador and Peru.

Results of the TCS, MYC model and Bayesian analyses using the ITS2 and *COI* data sets provides further strong support for the following species: *An. oswaldoi* s.s*.* from Brazil (Espírito Santo = type locality), Rondônia, Rio de Janeiro and São Paulo); *An. oswaldoi* A from Brazil (Mato Grosso, Rondônia, Amazonas) and Colombia (Amazonas); *An. oswaldoi* B from Colombia (Antioquia, Caquetá, Meta, Norte de Santander, Putumayo), Ecuador (Orellana) and Trinidad and Tobago (Valencia); and *An.* sp. nr*. konderi* from Colombia (Caquetá), Ecuador (Orellana) and Peru (Madre de Dios, Loreto) (Figures 1, 2, 3 and 4).

## Discussion

### *COI* and ITS2 sequence analyses

Based on mitochondrial *COI* and nuclear ITS2 sequences, this study presents clear molecular evidence that the Oswaldoi-Konderi Complex comprises at least five species: two previously determined (*An. oswaldoi* s.s. and *An. oswaldoi* B), as well as *An. oswaldoi* A and *An.* sp. nr*. konderi*, and *An. konderi* of Sallum defined herein for the first time. Phylogenetic reconstruction of *COI* and ITS2 sequences (both independently and concatenated) verified four distinct species, which concur with the MYC and TCS network models. Construction of genealogical networks using TCS are often used to infer evolutionary relationships at the population level; however, identification of independent networks that fall beyond the 95% parsimony connect limit are considered putative biological species [45]. At this threshold, four independent networks were generated corresponding to the four species listed above (Figure 1). In addition samples of *An. konderi* of Sallum, initially included as an outgroup in this study, not only represented a separate taxon but also fell firmly within the Oswaldoi Complex in phylogenetic treatments. This supports that *An. oswaldoi* and *An. konderi* comprise species complexes of very closely related taxa, which would benefit from being assessed concurrently.

Following this robust molecular delimitation, efforts were made to retrospectively identify samples documented in previously published studies, and relate these to geographical distributions and their regional malaria vector status.

### *Anopheles oswaldoi* s.s.

Prior to this study, the distribution of “true” *An. oswaldoi* s.s. had only been determined from Espírito Santo and São Paulo, Brazil [12]. Herein *COI* data also confirms its presence in the Brazilian States of Rio de Janeiro, Acre (GenBank: DQ784832-35) and Coari (GenBank: DQ784836-38) [15] (Table 4). Ruiz *et al*. [8,31] suggested a genetic distance threshold of 0.02 for species delimitation in *Nyssorhynchus* species. Despite the large geographic distance, our *An. oswaldoi* s.s. from São Paulo and Espírito Santo, Brazil (type locality, south of Brazil) grouped with Scarpassa and Conn’s *COI* sequences [16] from Rondônia State, in the north of Brazil (overall mean K2P distance of 0.010, range 0.002 to 0.015). Given its current distribution, it seems highly likely that *An. oswaldoi* s.s. could also be present in regions between these Brazilian states, including Mato Grosso, Goias and Minas Gerais, and perhaps even south into Bolivia or Paraguay.

**Table 4:** **Retrospective identification of published *COI *sequences of *An. oswaldoi *s.l. (DQ784827-DQ784851 **[16]**) and *An. konderi *s.l. (JF437965-JF437974 **[23]**) in relation to species determined herein, through sequence homology**

**Locality (Brazil)**	**GenBank accession**	**GenBank identification**	**osw s.s.**	**osw A**	**sp. nr. kon**	**kon of Sallum**
Acre, Sena Madureira	DQ784827	*An. oswaldoi*			98%	
	DQ784828	*An. oswaldoi*			98%	
	DQ784829	*An. oswaldoi*			99%	
	DQ784830	*An. oswaldoi*			98%	
	DQ784831	*An. oswaldoi*			98%	
	DQ784832	*An. oswaldoi*	99%			
	DQ784833	*An. oswaldoi*	99%			
	DQ784834	*An. oswaldoi*	99%			
	DQ784835	*An. oswaldoi*	99%			
Amazonas, Coari	DQ784836	*An. oswaldoi*	99%			
	DQ784837	*An. oswaldoi*	99%			
	DQ784838	*An. oswaldoi*	99%			
Rondônia, São Miguel	DQ784839	*An. oswaldoi*				99%
	DQ784840	*An. oswaldoi*				99%
	DQ784841	*An. oswaldoi*				99%
	DQ784842	*An. oswaldoi*				99%
	DQ784843	*An. oswaldoi*			100%	
	DQ784844	*An. oswaldoi*			99%	
	DQ784845	*An. oswaldoi*				99%
	DQ784846	*An. oswaldoi*				99%
	DQ784847	*An. oswaldoi*				99%
	DQ784848	*An. oswaldoi*				99 %
Pará, Moju	DQ784849	*An. oswaldoi*		99%		
	DQ784850	*An. oswaldoi*		98%		
	DQ784851	*An. oswaldoi*		99%		
Acre, Acrelândia	JF437965	*An. konderi*			100%	
Amapá, Macapá	JF437966	*An. konderi*				100%
	JF437967	*An. konderi*				100%
	JF437968	*An. konderi*				99%
Rondónia, Monte Negro	JF437969	*An. konderi*	99%			
Paraná, Porto Natal	JF437970	*An. konderi*	99%			
	JF437971	*An. konderi*	99%			
	JF437972	*An. konderi*	99%			
	JF437973	*An. konderi*	99%			
Paraná, Santa Helena	JF437974	*An. konderi*	99%			

**[osw s.s**.: *An. oswaldoi* s.s.; **osw A**: *An. oswaldoi* A; **sp. nr. kon**: *An.* sp. nr. *konderi*; **kon**: *An. konderi* of Sallum from Macapá, Amapá, Brazil].

There are no records to suggest that *An. oswaldoi* s.s. is a vector in the eastern Brazilian provinces of Espírito Santo, Rio de Janeiro or São Paulo. In the north-western Brazilian state of Rondônia, where *An. oswaldoi* s.s., *An. oswaldoi* A and *An.* sp. nr*. konderi* are present, de Oliveira-Ferreira *et al*. [46] and Klein *et al*. [47,48] reported low levels of *Plasmodium* infections in what can only be assumed to include a mix of *oswaldoi*-*konderi* lineages.

### *Anopheles oswaldoi* B

In this study, three ITS2 haplotypes [HX, HXI and HXII (Table 2)] were found to be identical to those reported as *An. oswaldoi* B from Putumayo, Colombia by Ruiz *et al*. [17,31]. These haplotypes shared 99% identity to published sequences from Ocamo, Venezuela (GenBank: AF055071) and Amapá, Brazil (GenBank: AF056318) (= Group II of Marrelli *et al*. [14]). Our analyses of both individual and combined *COI* and ITS2 data sets, grouped the above sequences into a single phylogenetic cluster and TCS network, confirming *An. oswaldoi* B as a separate species in the *An. oswaldoi* complex. This study increases the known distribution of *An. oswaldoi* B to include the Departments of Antioquia, Caquetá and Norte de Santander (Colombia), and the Province of Orellana (Ecuador) and Saint Andrew/Saint David (Trinidad and Tobago) (Figure 4).

*Plasmodium vivax* infection was detected in one molecularly confirmed *An. oswaldoi* B specimen (of 361 positive mosquitoes) collected in Putumayo, Colombia [13]. However *An. oswaldoi* s.l. has also been incriminated in Amapá, Brazil [49], as a secondary vector in Venezuela [50] and has been experimentally infected with *P. vivax* in Trinidad and Tobago [51], where only *An. oswaldoi* B has been identified to date. Correlation of these studies with the known distribution of *An. oswaldoi* B suggest the potential involvement of this species on malaria transmission is over a much wider region of northern and northeastern South America than originally realised.

Currently, the only valid synonym of *An. oswaldoi* is *An. aquacaelestis* Curry, originally described from Panama [52]. The original description differentiates *An. aquacaelestis* from *An. aquasalis*, and suggests that this Panamanian species is identical to the species present in Trinidad and Tobago. As *An. oswaldoi* B is the only species of the *An. oswaldoi* complex that has been detected in Trinidad and Tobago, it would be prudent to carefully study the type series of *An. aquacaelestis,* to ensure that our informally designated “*An. oswaldoi* B” is not in fact *An. aquacaelestis*, prior to assigning a new name.

### *Anopheles oswaldoi* A (designated herein)

Comparison of our *An. oswaldoi* A ITS2 sequences (as haplotypes HII-IX; Table 2) revealed high similarity with three in Marrelli *et al*. [4] and two in Sallum *et al*. [9]. Our Haplotype III from Tefé, Amazonas, Brazil and Amazon, Colombia, shares 99.8% identity with GenBank AF056317 from Amazonas, Brazil (= Group I of Marrelli *et al.*[14]) and varies by only a single base indel (−/T) at position 166. Our ITS2 haplotype HII from Mato Grosso, Brazil, is 99.4% identical to GenBank AF055068 from Acre, Brazil (= Group I of Marrelli *et al.*[14]) with base substitutions at positions 349 (C/A) and 427 (G/A). Our ITS2 haplotype HVI from Mato Grosso, Brazil, is 98.6% similar to GenBank AF055069 from Rondônia, Brazil (= Group I of Marrelli *et al.*[14]), and differs at only one base (position 370; G/A) from EU636802 [9]. Sallum *et al*. [9] described morphological differences in the male genitalia and ITS2 sequences of *An. oswaldoi* s.l. from Acrelândia, Acre, Brazil, compared to *An. oswaldoi* s.s. [12]. Our ITS2 haplotype HIV from Mato Grosso is identical to GenBank EU636809 from Acrelândia, Acre. These results clearly show that the unnamed species of *An. oswaldoi* s.l. described by Marrelli *et al*. [14] and Sallum *et al*. [9] corresponds to our *An. oswaldoi* A.

The *COI* sequences generated by Scarpassa and Conn [16] overlapped our barcode region by 404 bp. Comparison of their trimmed sequences with our data, revealed sequences from their “Group 3” specimens (GenBank DQ784849-51) from Moju, Pará, Brazil were 99% identical to those of our *An. oswaldoi* A, thus confirming that *An. oswaldoi* A is also present in Pará State (Table 4).

Drawing from our data and correlation with other published works [9,14,16], the known distribution of *An. oswaldoi* A now includes the Brazilian States of Acre, Amazonas, Mato Grosso, Pará and Rondônia, and the Colombian Department of Amazonas (Figure 4). Vector incrimination studies in Acre, Brazil determined *An. oswaldoi* as a local vector of malaria [53-56]. As the only confirmed species of the Oswaldoi Complex present in Acre, Brazil, it seems likely that *An. oswaldoi* A plays a role in malaria transmission in this state, and most probably throughout its distribution.

### *Anopheles* sp. nr*. konderi* (designated herein)

ITS2 haplotype HXIII was shared by a number of individuals from Madre de Dios and Loreto provinces in Peru, Orellana province in Ecuador, and in the Department of Caquetá, southern Colombia (Figure 4). These sequences showed high similarity (98-100%) to specimens identified as *An. konderi* s.l. from Amapá, Brazil [GenBank: JF437934-36, JF437926] [23], differing by only two transitions (G/A, position 361 and T/C, position 449) and a two base indels (at positions 264–265). We recognize that such intragenetic variation in ITS2 is not uncommon, having also been detected in *An. oswaldoi* A, *An. oswaldoi* B and other *Nyssorhynchus* species [9,57-59]. However, *COI* sequences from the same individuals analysed by Bayesian, MYC model and TCS network analyses confirm an independent sister cluster to the *An. konderi* of Sallum from Macapá, Amapá, Brazil, also included in our analysis. Both these taxa are unique with respect to a third *An. konderi* s.l. from Acre reported by Sallum *et al*. [9] (GenBank: EU636801).

The type locality of *An. konderi* is Solimões River, Coari, Amazonas, Brazil. However, in their recent redescription of *An. konderi,* Flores-Mendoza *et al.*[21] were unable to access material from this locality, using several other localities in Brazil instead. Some samples used in our molecular analysis were the same as those examined by Flores-Mendoza *et al.*[21], but as the identity of *An. konderi* is uncertain, we have designated this cluster as *An.* sp. nr*. konderi*.

The trimmed 404 bp *An. oswaldoi* “Group 2” *COI* sequences of Scarpassa and Conn [16] (GenBank: DQ784827-31 and DQ784843-44) are 98-99% similar to our *An.* sp. nr*. konderi* and 100% identical to GenBank: JF437965 [16] (Table 4). Correlation of our data with published works confirms the presence of *An.* sp. nr*. konderi* in three localities in Brazil (Acrelândia and Sena Madureira (Acre) and São Miguel (Rondônia)), and our data suggest that *An.* sp. nr*. konderi* may be allopatric in Madre de Dios and Loreto provinces in Peru, where *An. oswaldoi* s.l. has been confirmed as an efficient malaria vector [60-62].

The taxonomic status of *An. konderi* s.l. clearly needs to be reassessed. This and other studies [9,16,23] have provided evidence for at least three species in *An. konderi* s.l. Concerted efforts are needed to acquire material from the type locality in order to establish the identity of *An. konderi* s.s. before the systematics of closely related taxa can be properly understood. Given the data presented here, it is likely that the species discussed here will fall into a more comprehensive Oswaldoi-Konderi Complex in future.

## Conclusions

Through novel data and retrospective comparison of *COI* and ITS2 DNA data, evidence is shown to support three species within of *An. oswaldoi* s.l. (*An. oswaldoi* s.s., *An. oswaldoi* A and *An. oswaldoi* B), and at least three species in the closely related *An. konderi* s.l. (*An.* sp. nr. *konderi*, *An. konderi* of Sallum herein, and in Sallum *et al.*[9]). Determining the specific status and distribution of component members has allowed the circumstantial incrimination of *An. oswaldoi* A, *An. oswaldoi* B and *An.* sp. nr. *konderi* as malaria vectors. *Anopheles oswaldoi* s.s. has never been implicated in malaria transmission. Morphological studies of the species listed in the manuscript are now pertinent, to find reliable diagnostic characters, and to follow with the formal description and naming of the new species determined.

## Competing interests

The authors declare that they have no competing interests.

## Authors’ contributions

FRL and YML conceived the ideas; YML, DJP, RCW and MLQ obtained funding; FRL, MLQ, MAMS, CFM, DC, JA, JAO and RCW undertook fieldwork and/or donated samples; FRL, YML and MH carried out the molecular laboratory work; FRL, YML and RCW carried the data analysis and interpretation; FRL wrote the draft manuscript; YML, RCW, MAMS, DJP, MLQ, IDV, CFM, and JA revised the draft manuscript; FRL, RCW and YML carried out the final revision and submitted the manuscript. All authors have read and approved the final version of the manuscript.
